# Supplementary material for: The genome sequence of Carex sylvatica Huds., 1762 (Poales: Cyperaceae)
Source: Wellcome Open Res. 2026 Jun 2;11:332. [Version 1] doi: 10.12688/wellcomeopenres.26670.1 (PMC13560573; doi:10.12688/wellcomeopenres.26670.1)
Supplement: Peer review history

# Reviewer report: Reviewer response for version 1

**DOI:** 10.21956/wellcomeopenres.29371.r164343

**Published:** 2026-08-18

**Authors:** Ruslan Kalendar

**Affiliations:** 1 University of Helsinki, Helsinki, Finland

This Data Note reports a Darwin Tree of Life (DToL) reference genome for Carex sylvatica (Wood-sedge), a widespread woodland sedge of Britain and Ireland. A single Midlothian specimen (ToLID lpCarSylv1) was sequenced with PacBio HiFi on the Sequel IIe (11.25 Gb, 25×), scaffolded with Arima Hi-C, and assembled with hifiasm in primary mode followed by purge_dups, YaHS scaffolding and manual curation. The primary assembly spans 363.24 Mb in 50 scaffolds, with 99.79% assigned to 29 chromosomal pseudomolecules; organelle assemblies and an alternate haplotype are also deposited. Annotation is stated as forthcoming.

 The pipeline is an established and appropriate ToL workflow; the software versions and accessions are exemplary in their completeness, all data are openly deposited under PRJEB68036, and the Hi-C contact map (Figure 3) is clean and convincing. Carex is one of the largest angiosperm genera and is karyotypically among the most remarkable; therefore, an additional chromosome-level reference is genuinely valuable.

 My reservations are not about the validity of the data but about interpretation and internal consistency. In particular, roughly 17% of the estimated genome is absent from the assembly, which is nowhere acknowledged; the reported plastid genome length is markedly larger than any published Carex plastome; the collection coordinates place the specimen in the North Sea; and several curation and scaffold-count figures do not reconcile with the tables.

 1. The assembly spans 363.24 Mb, whereas GenomeScope2 estimates 433.89 Mb and flow cytometry gives 0.45 pg = 440 Mb. This is a deficit of approximately 70-77 Mb, approximately one-fifth of the assembly's own length, and the manuscript passes over it without comment.

 This bears directly on Reviewer 1's point about the 22.5% missing BUSCOs. He raises two possibilities (assembly incompleteness versus poales_odb10 divergence from Cyperaceae) and asks for both to be acknowledged. 

 2. Plastid. The reported length of 215.14 kb is roughly 27-31 kb larger than every published Carex plastome I am aware of: C. agglomerata 184,157 bp, C. myosuroides 185,609 bp, C. laevissima 188,029 bp, with Cyperus rotundus at 182,986 bp and Bolboschoenus planiculmis at 186,539 bp for wider Cyperaceae context. Cyperaceae plastomes are already large relative to most monocots, but 215 kb is an outlier even by that standard. The most likely explanations are an unresolved structural repeat or a duplicated inverted-repeat copy retained in the OATK output. Please verify the quadripartite structure and gene content, and either report the LSC/SSC/IR partition or flag the sequence as a provisional single-molecule representation.

 3. Table 2 reports 50 scaffolds spanning 363.24 Mb; the snail plot (Figure 5) and blob plot (Figure 6) both report 54 scaffolds and 365 Mb. Both reconcile neatly if the figures include the four organelle contigs (2.08 Mb); however, the reader must work this out separately. A footnote stating which counts include organelles would eliminate this ambiguity.

 4. Table 4 lists the benchmark as "S > 90%; D < 5%" against an observed S:68.7%, and the text nonetheless reports that the EBP metric of 6.C.Q65 meets the recommended 6.C.Q40 standard without qualification. Both statements are true in their own terms the EBP composite covers contiguity and consensus quality, not gene completeness but placed together they read as though every benchmark had been met. A single clause noting that the gene-completeness criterion is not met, cross-referring to the discussion requested in point 1, would resolve this.

 5. The Data availability section states that the genome "will be annotated" through the Ensembl pipeline. This is acceptable for a Data Note, but the Genome sequence report should state plainly that this version is unannotated, since the Abstract's silence on gene content may otherwise be read as an omission. Given Reviewer 1's concern, I would also encourage the authors to commit to reporting protein-mode BUSCO once annotation is released.

Are sufficient details of methods and materials provided to allow replication by others?

Partly

Is the rationale for creating the dataset(s) clearly described?

Yes

Are the datasets clearly presented in a useable and accessible format?

Yes

Are the protocols appropriate and is the work technically sound?

Yes

Reviewer Expertise:

bioinformatics, comparative genomics

I confirm that I have read this submission and believe that I have an appropriate level of expertise to confirm that it is of an acceptable scientific standard.

# Reviewer report: Reviewer response for version 1

**DOI:** 10.21956/wellcomeopenres.29371.r157342

**Published:** 2026-07-24

**Authors:** Sheng-Kai Hsu

**Affiliations:** 1 Cornell University, Ithaca, NY, USA

This Data Note reports a chromosome-level genome assembly for *Carex sylvatica* generated through the Darwin Tree of Life pipeline. The assembly is contiguous and of high base-level accuracy, the methods are thoroughly documented with tool versions and accessions, and all data are openly deposited. The work is a useful addition to the growing set of reference genomes for the British and Irish flora, and to genomic resources for *Carex*, a large and karyotypically distinctive genus. I only have one suggestion regarding gene space completeness that I would like the authors to acknowledge.

**Strengths.**

 The sequencing and assembly workflow (PacBio HiFi with hifiasm, purge_dups, Hi-C scaffolding in YaHS, ASCC decontamination, and manual curation) is the established and appropriate ToL approach. Base-level quality is excellent (combined QV 64.6; primary QV 65.4), contiguity is strong (contig N50 2.57 Mb, scaffold N50 13.81 Mb), and 99.79% of the assembly is assigned to 29 chromosomal pseudomolecules supported by a clean Hi-C contact map. Methods reporting is exemplary: software versions and sources are tabulated, pipelines are linked, and raw reads and both haplotype assemblies are deposited in INSDC databases.

**Suggestion**

 BUSCO completeness against poales_odb10 is C:75.2% [S:68.7%, D:6.6%], M:22.5%, which does not meet the single-copy (>90%) threshold listed in Table 4, with notably high missing gene content for a chromosome-level plant reference. This suggests potential assembly gaps extended to gene space. Another possibility is related to the suitability of the poales_odb10 lineage set for Cyperaceae: this set is heavily Poaceae-derived, and *Carex* is phylogenetically distant from the grasses and holocentric with extensively rearranged genome architecture, so some fraction of the "missing" BUSCOs may reflect reference-set divergence rather than assembly incompleteness. A few sentences acknowledging these possibilities (assembly incompleteness and ortholog-set mismatch) would help readers interpret the assembly for downstream use.

Are sufficient details of methods and materials provided to allow replication by others?

Yes

Is the rationale for creating the dataset(s) clearly described?

Yes

Are the datasets clearly presented in a useable and accessible format?

Yes

Are the protocols appropriate and is the work technically sound?

Yes

Reviewer Expertise:

Phylogenomics

I confirm that I have read this submission and believe that I have an appropriate level of expertise to confirm that it is of an acceptable scientific standard.
